# “Walking alongside:” collaborative practices in mental health and substance use care

**DOI:** 10.1186/1752-4458-8-55

**Published:** 2014-12-17

**Authors:** Ottar Ness, Marit Borg, Randi Semb, Bengt Karlsson

**Affiliations:** Centre for Mental Health and Substance Abuse, Faculty of Health Sciences, Buskerud and Vestfold University College, P.O. Box 7053, 3007 Drammen, Norway

**Keywords:** Community mental health care, Collaborative practices, Co-occurring mental health and substance use problems, Recovery, Shared decision-making, Action Research, Cooperative Inquiry

## Abstract

**Background:**

Although the importance of collaboration is well established as a principle in research and in theory, what it actually means for practitioners to collaborate in practice, to be partners in a collaborative relationship, has thus far been given less attention. The aim of this study was to identify key characteristics of the ways in which mental health practitioners collaborate with service users and their families in practice.

**Methods:**

This was a qualitative action research study, with a cooperative inquiry approach that used multi-staged focus group discussions with ten mental health care and social work practitioners in community mental health and substance use care. Thematic analysis was applied to identify common characteristics.

**Results:**

We identified three major themes related to practitioners’ experiences of collaborative practices: (1) *walking alongside through negotiated dialogues*, (2) *maintaining human relationships*, and (3) *maneuvering relationships and services.*

**Conclusions:**

It appears that even with the rich knowledgebase that has developed on the merits of collaborative relationships, it continues to be challenging for practitioners to reorient their practice accordingly. The findings of this study indicate that the practitioners focus on two types of processes as characterizing collaborative practice: one focusing on conversations among practitioners and service users and their families and the other focusing on management and control among health care providers, service sectors, and service users (i.e., inter/intra-system collaboration).

## Background

*“The manner of engagement – the way we develop a relationship with another person – influences the kind and quality of conversations that we can have with each other, and likewise the conversations we begin to have with each other will influence the kind and quality of our relationships.”* [1]

In the current mental health care context, there is a call for inter-professional collaborative relationships and practices to embrace the active participation of service users and their families in the process [2, 3]. At the core of these collaborative relationships are people’s competence and capability in listening, taking each other seriously, and respecting the perspectives of others concerning both the relationship and the partnership in which they are involved [1, 4–7]. Making collaboration succeed in mental health care requires a free flow of information and sharing of feedback among all parties so that they are on track with the changing intentions that often arise [8].

In the research literature that focuses on services and practices that keep the person at the center of decision-making, a number of essential principles are espoused. These include working with sometimes competing beliefs, values, and priorities; power and power balancing; engagement strategies; consistency of care delivery; relationship competencies; role blurring; and negotiated decision-making [6, 7, 9]. These are all complex concepts that are embedded in professional ideology and individual aspirations, and traversing these complexities can be considered key to determining good collaborative practices and dialogical conversations [1, 7].

There is robust research literature that shows that the most important foundation for collaborative practices and dialogical conversations in mental health care is the relationship between service users and practitioners [10–14]. This notion of collaboration has been described as the “client and the therapist forming a partnership against the common foe of the client’s deliberation pain” [15]. Thus, the concept of collaboration conveys a sense of trust, flexibility, teamwork, partnership, cooperation and working together toward shared goals [16–18]. Combining such a relationship with a recovery orientation further involves a reorientation from the professional being an expert on other peoples’ lives towards supporting individuals in their own ways of managing problems and struggles [16, 19].

Recovery is related to person’s own efforts and work in getting on with life and creating a life in a community, in spite of different life struggles (such as mental health and substance use problems), with and without help from professionals [16]. Recovery-oriented practices are related to what professionals offer and do to help persons in recovery. This can be support on a personal level (i.e., helpful relationships) and on the system level (i.e., lobbyist, human rights, and anti-stigma work [16, 18, 19]).

From first-person accounts of lessons learned in recovery, it is clear that recovery processes take place in everyday life [20–23]. Research shows that recovery processes are facilitated by having a meaningful life with meaningful activities [18, 24, 25], focusing on strengths and future orientation, and re-establishing social life and supportive relationships [20, 26, 27]. Recovery literature also shows that barriers to recovery processes include the lack of tailored help and the need to navigate in complex systems and uncoordinated services [27]. Persons in recovery value practitioners who convey hope, share power, are available when needed, are open regarding the diversity in what helps, and are willing to stretch the boundaries of what is considered the “practitioner’s” role in the services [19]. Recovery-oriented practitioners are those who have the courage to address the complexities and the individuality of persons’ own change process and use their professional skills and expertise in a collaborative partnership with the person.

Although the significance of collaboration is well established in theory in the mental health field [5, 28], what it means to collaborate in practice, to be partners or to be mutually involved in a partnership has thus far been given less attention in the literature. In addition, it is notable that even with the rich knowledgebase we have on collaboration and recovery, it continues to be challenging for practitioners to practice accordingly. Although, in general, practitioners want to be collaborative, their practice often reveals the contrary [3, 7, 27].

The purpose of this article is to provide practitioners’ own perspectives on collaborative practice in working together with young adults with co-occurring mental health and substance use problems and their families. The research question for this study was: *How do mental health care practitioners understand and describe collaborative practice with service users who are young adults with co-occurring mental health and substance use problems and their families?*

## Methods

### Design

The overall design of this research is action research with a cooperative inquiry perspective [29]. Cooperative inquiry refers to a variety of approaches, and it is regarded as particularly appropriate in action research based on participatory philosophy [30]. The research questions were identified by involving practitioners within the mental health, substance use, and child and family services in a municipality in Norway. The design was longitudinal, qualitative, and cooperative. The researchers and participants in the role as co-researchers worked collaboratively in identifying problems, deciding on themes for inquiry, selecting a research design, and designing projects for implementation [31]. A person with experience as a mental health service user participated as a moderator along with the researcher in the focus group discussions and was involved as a co-researcher in this participatory research project.

As a part of the action research methodology, we established a “competence group” to work with the research team in all stages of the study. The competence group consisted of two family members, two service users, and three practitioners from the municipality, but they were not participants in the focus group discussions. Inspired by the concept of participatory research [32–35], this group was involved in developing the research project in detail, e.g., working out the interview guides and inclusion criteria, conducting data analysis, and in participating in ongoing planning and discussions throughout the entire study. The competence group was a part of the entire research project and met four times annually throughout the implementation of the project. The report in this paper is from one part of this project.

### Data collection

In the present study, multistage focus group discussions were adopted to engage the practitioners actively in the research. The multistage focus group discussion is characterized by exploring a certain theme or phenomenon through several group discussions, and it is described by Hummelvoll [36] as inquiring into knowledge dialogues emerging from experiential material.

Three monthly focus group discussions were audiotaped, from which material was transcribed verbatim. The focus group discussions were held with the participants as the co-researchers, and the first and third authors attended all discussions. The duration of the focus group discussions was usually 1.5 to 2 hours. The focus group discussions were moderated by the researchers, who introduced the themes for discussion regarding ideas and practices about collaborative practice experienced by the participants in their work. Summarized notes of the transcriptions for each meeting were shared with the participants at the beginning of the subsequent meeting for feedback and to provide a context for a deeper conversation on collaborative practice. In this way, it was possible both to articulate the participants’ professional knowledge and to elevate this experience-based knowledge to a higher level of abstraction. The open life world approach incorporated into multi-staged focus group discussions provided the perspective that the development and understanding of processes of collaborative practice would emerge from the professionals’ experiences.

### Participants

Participants for this study were recruited from the Mental Health, Substance Use and Child- and Family Services in a municipality in the Eastern part of Norway. The inclusion criterion was that they had at least two years of experience in working with young adults with co-occurring mental health and substance use problems. There were 8 mental health nurses and 2 social workers, all with further education in mental health care, substance use care, and family therapy. In the three focus group discussions, six participated three times, four participated two times, and two participated only once. Absence was due to clinical responsibilities and personal illness.

### Thematic analysis

Following the aim of this study, the transcribed text from the multi-staged focus group discussions was analyzed using thematic analysis [37, 38]. The first author conducted the initial data analysis by reading the transcripts to become familiarized with the data, noting initial thoughts, ideas, and emerging themes. Subsequently, the material was coded using the research question as a guiding question. The initial ideas and the emerging themes were then condensed, interpreted, labeled, and categorized and subsequently condensed into a coherent text and merged with the preliminary themes from the first reading. Meaningful elements, such as quotes and descriptions of the emerging themes, were identified, listed, and collated and then sorted into seven tentative categories (building a trusting and supportive relationship, creating supportive arenas for collaboration, relational processes, structural and organizational framework for collaboration, participants’ own goals and negotiating a way forward, focusing on everyday life contexts, and collaborative involvement with the community). The data were examined several times to complete the categories into overarching themes. The competence group was also involved in discussing the analysis process and preliminary findings with the researchers. The first author presented the preliminary findings to the members of the competence group in one group meeting. The members of the competence group read summaries of the preliminary findings. This gave them an opportunity to comment or share ideas on how the preliminary findings might be understood and possible implications and relevance regarding collaborative practices based on the practitioners’ perspectives within the municipality. The internal validity of the findings was enhanced by the second, third and fourth authors’ discussions of the analysis process and findings with the first author. They also contributed to writing the text that described the themes and the subsequent discussion.

In our thematic analysis, we identified three overarching themes about practitioners’ experiences of collaborative practices in mental health and addiction care: (1) *walking alongside through negotiated dialogues*, (2) *maneuvering relationships and services*, and (3) *maintaining human relationships.*

### Ethical approval

The study was conducted in accordance with The Norwegian National Committees for Research Ethics. Ethical approval to conduct the study was granted by the Norwegian Social Science Data Services (NSD). After a complete description of the study to the participants, written informed consent was obtained. Confidentiality was assured for participants.

## Results

### Walking alongside through negotiated dialogues

The participants in this study described how collaborative practices with young adults with co-occurring mental health and substance use problems and their family members involved “*walking alongside*” them. They described these partnerships as negotiated dialogues towards a mutually agreed upon destination. They said that in establishing collaboration with a service user, it was crucial to take his or her life situation, hopes, and dreams as the starting point and then discuss good ways of working together from there. As one of the participants said: *In a fruitful collaboration, it is not about just giving information to each other but negotiating a way of working together so that we can have a joint understanding of how to proceed with the work together.*

Participants discussed that walking alongside young adults and their family members also involved building (or negotiating) a good relationship with them. They described that when they work together with service users, it is “*very important not to take over the service users’ life but be with them and help them with what they want*.” In this way of walking alongside service users, they emphasized that they have to be flexible in their way of working; they need to be available for the service users and their families. The participants also highlighted in the discussion that it is important to inform service users about their civil and human rights and about the nature of the assistance that they can receive from the service providers so that the persons can make their own choices. As one of the participants said: “*It is important for the service users that we meet to experience that we are available and flexible (…) you can’t just sit in your office; you need to meet the service users where they are, and that can change quite quickly*.”

Participants also talked about how walking alongside service users involves supporting them in their everyday life challenges. As one said: “*I feel that my work is mostly about helping service users with their everyday life, school issues, work, activities, having a place to live, etc. And then I have to go with them and not impose my way of having an everyday life*.”

Finding ways of working together in this way, by negotiating the way forward with persons, requires “*respect for the persons’ integrity and life*,” as one participant said. However, *“this is not always easy to do,”* another said, particularly due to inflexible work conditions and huge caseloads for the practitioners. They said that it was not easy to be patient when following the service users’ needs on a moment-to-moment basis. This was because they often had a sense beforehand of what they thought was important for the service users to do to in their life to get better. Putting the service user first and following his or her lead required putting aside the practitioners’ own sense of what may have been more useful.

Another topic they talked about when they were working with the different parties in collaboration around, and with, a service user was the contract of confidentiality. They emphasized the importance of always negotiating the contract of confidentiality with services users and family members. This was a challenging task for them. They said that one day, the service user could say that the practitioners could talk about sensitive issues with other practitioners or with family members, but the next day, he might withdraw this consent. This could be experienced as frustrating by the practitioners and for family members who wanted more collaboration and involvement from the practitioners with their young adults. The participants agreed that they needed to get better in involving family members in the services, but this requires a negotiation with both service users and family members about how the involvement will take place. One of the participants said: “*You also need to give time, be available and flexible towards family members to create a safe environment for them to be involved in collaboration with the service users*.” As a response, another participant said: “*We are trying to collaborate a lot with family members, but there are always dilemmas as some of the service users grade their contract of confidentiality, but you need to be flexible, available, and creative to negotiate a good collaboration with them all*.”

Participants emphasized, in the group discussion, that when they walked alongside the service users and made sure not to go too fast, it helped them to develop a trusting relationship, as long as they always had the service user’s goals at the forefront of their work and negotiated ways to go on together.

### Maintaining human relationships

Another issue explored in the focus groups was about not giving up and maintaining human relationships. This requires practitioners to have continuity and time to “*be there, over time together with people*,” as one said. It is important that they, as practitioners, not give up on people, and “*You need to give more than people expect*,” as one said. For example, as a practitioner and a contact person, you approach service users in a friendly manner based on their own (practitioners’) initiative rather than waiting for the service users’ initiative to meet. One participant reflected upon this: “*I have experienced that it is very important that you drop by, visit them, call them, even if they haven’t shown up for the last three appointments*.” The participants responded to this as important because the practitioners learned from service users that they change contact persons so many times that they sometimes give up because they have to tell their story so many times to new practitioners. One said: “*It is not quality time only that matters but that you are persistent over time so that the service users know you are available for them*.”

Maintaining human relationships was how the practitioners took user involvement seriously and concretely in their everyday practice. This involved seeing the person as a unique individual and not giving up. One of the participants said: “*We are working with people who are different; we have to be there to insist on people’s strengths and possibilities. It is human beings we are helping; we need to not give up on them*.” However, the participants said that they have to help service users to see their own strengths and possibilities; it is “*important that it is not the practitioners’ ideas of how to live a good life that is the measure; it is the persons’ own thoughts, hopes and dreams that have to be in focus*.”

Participants described, in the group discussions, how collaborative practices are about not giving up, being persistent, and insisting on human relationships even if the systems in which they worked were fragmented and difficult to understand for persons requesting their support.

### Maneuvering relationships and services

Practitioners discussed collaborative practices as involving maneuvering relationships and services. Because the services in the municipality (and elsewhere in the Norwegian health care) are organized in quite a fragmented manner, the participants agreed that “*there are so many actors that you are going to collaborate with—the service user, family members, schools, general practitioners, social services, colleagues, other services etc.—that I find it very difficult to maneuver in this myriad*.”

In the focus group discussions participants were also concerned about the myriad bureaucracies that persons struggling with mental health and substance use problems meet when they ask for help. These multiple systems create situations in which practitioners have to spend as much time maneuvering service users through these systems as they do in helping them with their everyday life. As one participant said:

*I get frustrated on behalf of the persons when I know what goals he has for his life, but when he uses our services, he or she will meet a bureaucracy and system. So what we have to do is to work with the person in parallel with the system so that the person does not get lost; we almost need to do as much motivation work so that the person does not get tired when meeting this system…as much as we offer practical help and helping conversations.*

Participants described how all of the different aspects of bureaucracy, documentation, and fragmented services led to less time to collaborate directly with the persons asking for help. One participant said: “*Because of all of the things we are expected to do is very time consuming … the time I use with the service users now, is about one third less than before… the time goes instead to administration, writing different reports etc.*” Because of this, participants focused on the importance of service users having one coordinator following them. They also mentioned that it is important that the practitioners have an overview of all the activities and providers that service users can access within the municipality.

Participants also reflected upon, in the discussion, how they experienced practitioners and managers on various levels, who seemed to have more loyalty to the system than to the service user as a human being. As one participant said: “*We need to remind ourselves that the system needs to take care of person’s wishes and needs, not the opposite*.” Another participant asked: “*There are so many competent practitioners and services in the municipality, but are we using the competencies most efficiently*?”

Another important aspect of maneuvering all the relationships and services was the need for flexible working approaches and a flexible framework within which to work. Participants said that it is very important to have good managers that really understand what it is like to collaborate with struggling persons and that there is not one uniform way of helping them. As one explained: “*You need good managers that support your work and really know in practice what it is like doing this kind of work*.” However, participants were concerned that their managers had too much to do with administration and therefore did not have the time to see or support their work.

Participants also discussed the importance of knowing other practitioners in the different service provisions personally. They claimed that this enhanced their way of collaborating and maneuvering in the system. As one said:

*It is very important that we meet other practitioners in the other services on a regular basis so that we can know what we can do together, and then it is easier to know where to ask for the different competencies you need when giving good help to the service user.*

Participants discussed the challenges a fragmented system poses, particularly in relation to collaborating with so many different persons and services in the municipality. Thus, maneuvering relationships and systems are important in collaborative practices.

## Discussion

We present our discussion around two somewhat different processes of collaboration based on our findings: (1) collaborative practices with service users and their families and (2) collaborative practices on system levels.

### Collaborative practices with service users and their families

Our findings suggest that collaborative practices with service users and families mean “walking alongside” them. This requires practitioners need to start with service users’ own goals, hopes, and dreams and negotiate ways of working together from there. Because practitioners cannot “recover” people directly, services need to offer the preconditions of helping relationships that foster recovery through enhancing persons’ access to opportunities and support [39]. A recovery-oriented practitioner who works in partnership with service users is able to walk alongside them and support their life processes through helping relationships and conversations. Relationships and conversations in mental health and substance use care are inseparable and influence each other [7]. The person-centered engagement that practitioners use in developing a collaborative relationship influences the type and quality of conversations that they can have with each other. Likewise, the conversations practitioners begin to have with a person will influence the type and quality of their relationship [1, 40].

For example, the service user and the practitioner each bring a unique knowledge and “expertise” to the relationship: Persons with co-occurring problems have insights and experiences relevant for themselves and their lives, and practitioners have expertise related to treatment processes, service provisions, and activities. In addition, they bring their personal knowledge and life experiences and can create space for collaborative relationships and dialogic conversations. They jointly develop expertise and knowledge that is an inter-subjectively shared form of knowing from their respective perspectives. In this way, they can negotiate dialogues and relationships forward. The focus, however, is on identifying and nurturing the service user’s expertise and strengthening his or her ability to handle everyday life. In this way, a service user also helps to orchestrate his or her own help, sharing the decision-making in all aspects of care. If practitioners have an opinion, for instance, about the participants of a treatment team, they should express it, give the reason for it, and encourage discussion. At the same time, however, they should respect a persons preferences and negotiate their way forward from there [1, 7]. This type of shared decision-making between all stakeholders is essential in collaborative practices [41]. This is the ethical imperative that shared decision-making and collaboration rest upon [42].

Sustaining human relationships was another theme that was central in revealing the processes in collaborative practices. This means that the practitioners upheld the idea that service users are first and foremost human beings struggling with different mental health and substance use problems [43, 44]. Persons with these life challenges can feel ashamed, try for long periods of time to hide their difficulties, and often feel stigmatized [43]. Stigma and discrimination have a troublesome effect on many peoples’ lives, diminishing their sense of hope and self-esteem [45]. They can cause them to feel looked down upon and distrusted or perceived as difficult to help, uncooperative, and unmotivated [27]. The focus of the collaborative practices revealed in our findings suggests a need to recognize the importance of context and relationships. This represents a change from a focus on identifying disease to emphasizing peoples’ lives and paying attention to service users’ everyday life, activities and work, the partnerships being developed, the sense of belonging, and the person’s home [46, 47]. Human beings need continuity and security, and in helpful relationships, autonomy and flexibility are essential ingredients. Our findings suggest that the processes of collaborative practices involve assuring equal human relationships, which can evolve into a focus on collaborative relationships and dialogue over time [1, 3].

### Collaborative practices on system levels

Another aspect of collaborative practices that emerged in the data is regarding inter-system collaboration. The key idea is that the practitioners become “advocates” for service users and families working with the parties in the service system and coordinating with other service providers on different system levels. The way the practitioners do this in practice is by maneuvering all the relationships and services that are available for the service users and families who need support.

Organizational, social, and cultural contexts are the sources that shape and influence collaborative practices [19, 48]. The complexity of mental health issues and the diversity of recovery processes must to be considered within the multifaceted contexts where both service users’ experiences and service provisions are couched [19]. Practitioners and managers of the services need to be mindful that collaboration does not take place in a vacuum. As with any relationship, it is influenced by many factors, such as the attitudes of what collaboration means, environmental conditions, economic structures, and the arenas in which meetings between mental health care practitioners and service users take place. A fragmented health care system without an integrative coordination process in place can result in duplication of services, missing critical services, or confusion that results primarily from what Le Boutillier *et al.* [49] call “competing priorities.”

Practitioners working in such a system have to struggle to maintain collaborative practice with service users and their families, often having to address forces within the service system such as the inability to sustain continuity in service provision or a lack of system-wide support. Practitioners report that they do not get the contextual and leadership support required to walk alongside persons having life struggles. This means that practitioners see competing priorities across the different layers of the health care system. For example, the critical aspect of “helpful help” voiced by service users is for practitioners to have the courage to address the complexities and the individuality of persons’ own change processes and the ability to use their professional skills and expertise in a collaborative partnership with service users within the system of care. However, practitioners report that they experience tension between what they know and experience as helpful for persons and the way the services are organized and developed, which does not support the needed autonomy and flexibility.

Practitioners in this study handled these issues by stretching some of the system rules and maneuvering the system on behalf of service users and families. Practitioners have the responsibility to negotiate with various levels of the health care system and navigate passages for services users and families to protect them from harmful fragmentation and try to offer the best possible care.

### Methodological limitations

One limitation of this study lies in the difficulty of distinguishing between what is practiced and what is believed to be ideal in focus group discussions. A good alternative approach would be to do a participatory fieldwork study to describe what practitioners actually do in collaborative practice. Another limitation is the imbalance in knowledge, perspectives, strategy and aims between researchers, practitioners, service users and family members. How democratic is the research process when well-educated researchers invite service users and family members to collaborate on a research project? How equal are the collaborative relationships in the different steps of such a research project and analysis of data? The last limitation is that only practitioners participated in these focus group discussions; it would be interesting to invite both service users and family members to elicit similarities and differences in experiences of collaborative practices.

## Conclusions

Although the significance of collaboration is well established in theory and practice in the mental health field [5, 28], what it means to collaborate in real settings is not always clear. What being partners actually involves has been given less attention in the literature. It is worth noting that in spite of the rich knowledgebase on collaboration and recovery, it continues to be challenging for practitioners to practice accordingly. In addition, practitioners experience that the legal and administrative health and welfare systems that are supposed to support collaborative and user involved partnerships are often felt to be barriers to collaboration and recovery. Business models and measures characterize the introduction of the neo-liberal New Public Management (NPM) into in today’s health care services [50]. Stamsø [51] claims that health care practitioners feel that there is more focus on efficiency and results than on the quality of services.

Ness *et al.* [27] propose two critical, interconnected components in collaborative practice: (a) collaboration among practitioners, service users, and families/networks (i.e., help and support processes) and (b) collaboration among healthcare providers, service sectors, and service users (i.e., system processes). The findings in this study indicate that the practitioners apply both processes in their ways of trying to help and support people in recovery, one focusing on communication and relationships and the other focusing on system management and system control rather than collaboration.

Practitioners view subtle communication skills to be aligned with service users in their roads to recovery and maintaining human relationships with them. The practitioners also made use of their system knowledge in order to support the person they worked with. It was often necessary to give priority to the person’s needs and put them above the system demands. Professional competencies encompass the coordination of two components of collaboration for best outcomes in service users. There is a need to examine how this coordination actually is developed and maneuvered in future studies. Furthermore, for collaboration to happen in the second component (i.e., system-level collaboration), it seems critical to have organizational structures and processes that promote collaboration among service providers, service sectors, and service users. Without an established structure for collaboration in this arena, practitioners are more likely to resort to managing, manipulating, and controlling system factors on behalf of service users rather than engaging in true collaborative processes involving service users. In addition, one should think that managing, manipulating, and controlling could also be present in the relationship between practitioners and service users in the difficulties in practicing collaborative practices.

## Authors’ information

ON is a family therapist and PhD. He works as an Associate Professor in mental health care at Buskerud and Vestfold University College, Norway.

MB is an occupational therapist and PhD. She works as a professor in mental health care at Buskerud and Vestfold University College, Norway.

RS is a sociologist and MSC. She has lived experience from mental health problems. She works as researcher in mental health care at Buskerud and Vestfold University College, Norway.

BK is a nurse, family therapist and PhD. He works as a Professor in mental health care at Buskerud and Vestfold University College, Norway.
